# Adaptation of *Saccharomyces* Species to High-Iron Conditions

**DOI:** 10.3390/ijms232213965

**Published:** 2022-11-12

**Authors:** Raquel Sorribes-Dauden, Tania Jordá, David Peris, María Teresa Martínez-Pastor, Sergi Puig

**Affiliations:** 1 Departamento de Bioquímica y Biología Molecular, Universitat de València, Doctor Moliner 50, 46100 Burjassot, Spain; 2 Departamento de Biotecnología, Instituto de Agroquímica y Tecnología de Alimentos (IATA), Consejo Superior de Investigaciones Científicas (CSIC), Agustín Escardino 7, 46980 Paterna, Spain; 3 Section for Genetics and Evolutionary Biology, Department of Biosciences, University of Oslo, Blindernveien 31, 0371 Oslo, Norway

**Keywords:** iron toxicity, yeast, *Saccharomyces* genus, adaptation, evolution, *CCC1*, oxidative stress, biodiversity

## Abstract

Iron is an indispensable element that participates as an essential cofactor in multiple biological processes. However, when present in excess, iron can engage in redox reactions that generate reactive oxygen species that damage cells at multiple levels. In this report, we characterized the response of budding yeast species from the *Saccharomyces* genus to elevated environmental iron concentrations. We have observed that *S. cerevisiae* strains are more resistant to high-iron concentrations than *Saccharomyces* non-*cerevisiae* species. Liquid growth assays showed that species evolutionarily closer to *S. cerevisiae*, such as *S. paradoxus*, *S. jurei*, *S. mikatae*, and *S. arboricola*, were more resistant to high-iron levels than the more distant species *S. eubayanus* and *S. uvarum*. Remarkably, *S. kudriavzevii* strains were especially iron sensitive. Growth assays in solid media suggested that *S. cerevisiae* and *S. paradoxus* were more resistant to the oxidative stress caused by elevated iron concentrations. When comparing iron accumulation and sensitivity, different patterns were observed. As previously described for *S. cerevisiae*, *S. uvarum* and particular strains of *S. kudriavzevii* and *S. paradoxus* became more sensitive to iron while accumulating more intracellular iron levels. However, no remarkable changes in intracellular iron accumulation were observed for the remainder of species. These results indicate that different mechanisms of response to elevated iron concentrations exist in the different species of the genus *Saccharomyces*.

## 1. Introduction

Iron is a necessary redox cofactor for many key biological processes, like DNA replication and repair, amino acid and protein biosynthesis, lipid metabolism and cellular respiration (reviewed in References [1,2,3]). Despite being especially abundant in the Earth’s crust, in oxidative environments, iron easily switches from Fe^2+^ to Fe^3+^, with solubility and bioavailability that are remarkably low at physiological pH. Thus, in humans, iron scarcity often leads to iron deficiency anemia, which causes fatigue and impairs cognitive development and immunity. Iron limitation is also an agronomic problem since low iron availability decreases crop yield and quality (reviewed in Reference [4]). Moreover, competition for iron between microbial pathogens and their hosts modulates both their virulence and the host’s capacity of response (reviewed in Reference [5]). Thus, organisms have developed different mechanisms to overcome iron shortage. In the yeast *Saccharomyces cerevisiae*, in response to iron deficiency, the transcriptional factors Aft1 and Aft2 activate a group of genes known as the iron regulon. These genes codify proteins that are involved in iron acquisition through iron-reductive uptake or the capture of siderophores, mobilization from intracellular reservoirs, such as the vacuole, recycling, and metabolism remodeling in order to optimize the use of the scarce iron (reviewed in Reference [6]). However, iron in excess is also harmful to cells since it can be involved in Fenton reactions that generate radical oxygen species (ROS), which induce damage to lipids, proteins, and nucleic acids [7]. Nonetheless, iron toxicity can even occur in anaerobic conditions, and other mechanisms, such as the mismetallation of enzymes or the activation of sphingolipid signaling have been proposed to mediate toxic iron effects in yeast [8,9,10]. In the presence of high iron levels, the *S. cerevisiae* transcriptional factor Yap5 activates the expression of *CCC1*, *GRX4*, and *TYW1* genes. The main factor responsible for yeast iron detoxification in Ccc1 is an iron transporter localized at the vacuole membrane that imports iron from the cytosol. Grx4 is a monothiol glutaredoxin that binds iron-sulfur (Fe-S) clusters and is involved in Aft1 deactivation. Tyw1 is a cytosolic Fe-S cluster-containing enzyme involved in the synthesis of the modified nucleoside wybutosin. Both Grx4 and Tyw1 have been described to play a key role in iron buffering in the cytosol [11,12]. Iron overload also limits iron acquisition through the downregulation of the low-affinity Fet4 and high-affinity Fet3-Ftr1 cell surface iron transport systems (reviewed in Reference [6]). In addition, *S. cerevisiae* activates several genes involved in oxidative stress response, as *TRX2*, *SOD1*, *TSA1* and *YAP1*, when iron is present at high levels [10]. The above-described responses to low and high iron are generally conserved in the *Saccharomyces* genus, and in a broader sense, the iron uptake and storage systems are also conserved in fungi, with differences in the transcriptions factors involved (Aft1 vs. iron responsive GATA factors), or the ability to produce siderophores [5,13,14]. Recent data have shed light on several biological mechanisms that have permitted the adaptation to iron scarcity in fungi, including horizontal operon transfer of bacterial genes involved in siderophore biosynthesis, loss of function of Ccc1 and Yap5, and the increase in iron uptake, which has been proposed to occur through mutations in Aft1 or the iron uptake system, or by alterations in the expression of other iron homeostasis genes [13,14,15]. On the other hand, the adaptation to high iron levels requires changes in storage and energetic metabolism, upregulation of oxidative stress genes, and iron uptake avoidance [13,16]. Furthermore, in the last two decades, the number of isolated, phenotyped, and sequenced strains among the *Saccharomyces* genus has unprecedently risen, consolidating this genus as a model to explore adaptation and evolution [17,18,19]. Previously, we analyzed the response to variations in the iron levels of a wide selection of *S. cerevisiae* strains, from a diversity of origins. In this report, we have extended the study of the response to excess iron to representative strains of the main available *Saccharomyces* populations. We characterized their growth, oxidative state, iron accumulation, and expression of iron homeostasis genes. Our results confirm that the main mechanism to avoid iron toxicity in the *Saccharomyces* genus is to diminish iron incorporation, but other mechanisms must be considered, since iron sensitivity is found in species with low iron accumulation.

## 2. Results

### 2.1. Characterization of Iron Resistance and Accumulation in the Saccharomyces Genus

**Figure 1:**
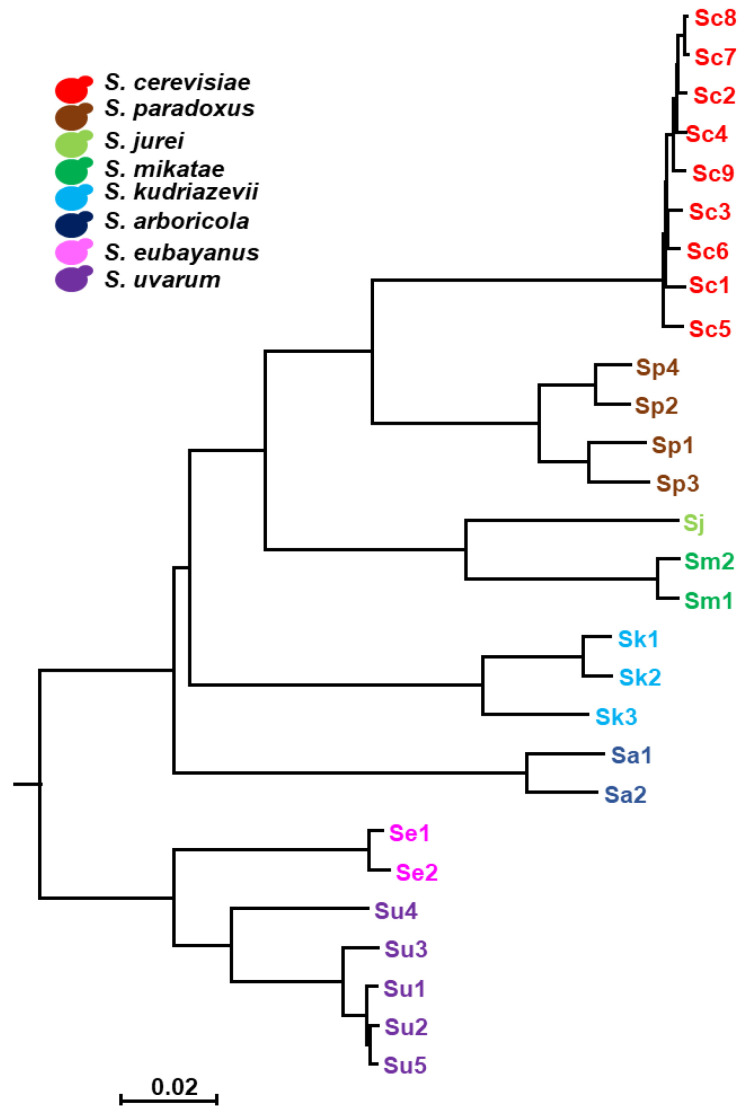
The phylogenetic tree of the *Saccharomyces* strains used in this study. Neighbor-Joining (NJ) tree using the (100 − ANI)/100 values as distances for reconstruction. The scale bar represents (100 − ANI)/100. The color code assigned to each *Saccharomyces* species in this study are: *S. cerevisiae* (Sc), red; *S. paradoxus* (Sp), brown; *S. jurei* (Sj), light green; *S. mikatae* (Sm), dark green; *S. kudriavzevii* (Sk), light blue; *S. arboricola* (Sa), dark blue; *S. eubayanus* (Se), pink; and *S. uvarum* (Su), purple.

In a previous report, we analyzed the response of a set of *S. cerevisiae* strains, from a wide diversity of origins, to excess iron, and selected a group of strains either resistant or sensitive to elevated concentrations of iron [13]. In the present study, we aimed to expand our knowledge of how yeast cells respond to high iron to other species of the *Saccharomyces* genus, for which we selected 28 strains belonging to eight different *Saccharomyces* species (*S. cerevisiae*, *S. paradoxus*, *S. jurei*, *S. mikatae*, *S. kudriavzevii* and *S. arboricola*) and challenged them with high extracellular iron concentrations (Table 1 and Table S1, and Figure 1). Nineteen *Saccharomyces* non-*cerevisiae* strains were selected as representative of the genus, while nine previously characterized *S. cerevisiae* strains were used as references [13].

To characterize how these strains respond to excess iron, we grew them at 20 °C on the SC liquid medium with increasing ferrous ammonium sulfate (FAS) concentrations, and growth was followed through OD_600_ measurements every 30 min, as described in the Materials and Methods. The chosen temperature was 20 °C because it provides a similar growth rate for all the *Saccharomyces* species studied [20]. The area under the growth curve at a particular iron concentration (AUC_Fe_) was obtained and normalized to the AUC for the same strain when cultivated without iron to obtain the fractional area (Fa = AUC_Fe_/AUC). Finally, Fa was plotted versus log_10_ of iron concentration (Figure S1). In all cases, the representation of the Fa vs. log_10_[FAS] fitted to a sigmoid curve, indicating that iron-susceptibility is non-lineal in the *Saccharomyces* genus.

**Figure 2:**
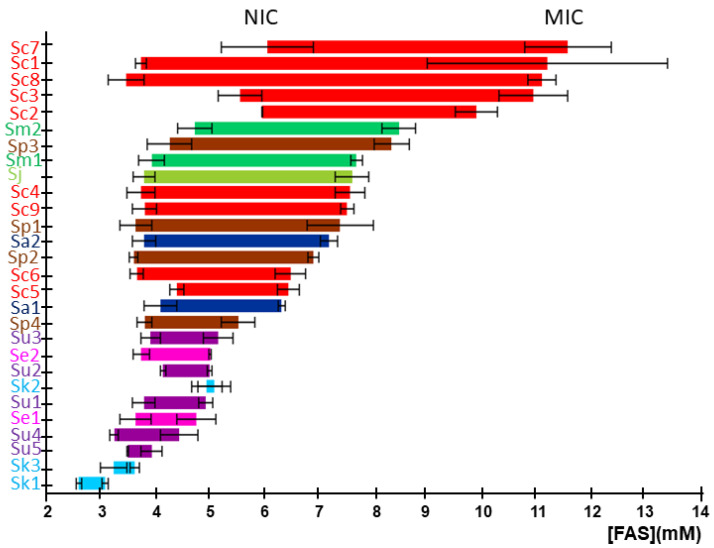
Growth inhibitory effect of iron on *Saccharomyces* strains. Horizontal segments represent the range of FAS concentrations between NIC (left-end) and MIC (right-end), for each yeast strain. Data and error bars represent the average and standard deviation of three independent biological replicates. The colors of segments and strain abbreviations are species-dependent according to Figure 1.

The non-inhibitory concentration (NIC) and the minimal inhibitory concentration (MIC) were obtained for each strain from their corresponding Fa plots (Figure 2). These values delimit the concentration range of iron with inhibitory effects on the growth for each particular strain. The *S. cerevisiae* strains Sc2, Sc3, Sc7, and Sc8 showed high NIC and MIC values, supporting the results of our previous study, performed in solid media at 30 °C, which characterized these strains as highly iron-resistant [13]. However, *S. cerevisiae* strains Sc6, Sc5, and Sc4, previously identified as iron-sensitive [13], displayed lower values of NIC and MIC. The Malaysian UWOPS03-461.4 strain (Sc1), characterized as iron-sensitive [14], presents a low value for NIC, but a MIC value higher than expected. Finally, the laboratory strain BY4743 (Sc9) displayed an intermediate iron sensitivity (Figure 2). A general observation shows that most strains possess NIC values around 3.5 mM, indicating that species start being affected by iron at similar iron concentrations, but only some of them are also resistant through a wider range of iron levels. Thus, ΔMIC-NIC range or MIC value could be used in most cases to classify strains according to iron sensitivity. Regarding the comparative resistance between species, the highest resistance is found in *S. cerevisiae* strains, followed by the *Saccharomyces* species that are more closely related to *S. cerevisiae*, including *S. mikatae*, *S. jurei*, and *S. paradoxus* (except for strain Sp4), which are highly or moderately resistant to elevated iron concentrations. Conversely, *S. eubayanus*, *S. uvarum*, and *S. kudriavzevii* are the most sensitive species, while *S. arboricola* behaves between those two groups of species.

**Figure 3:**
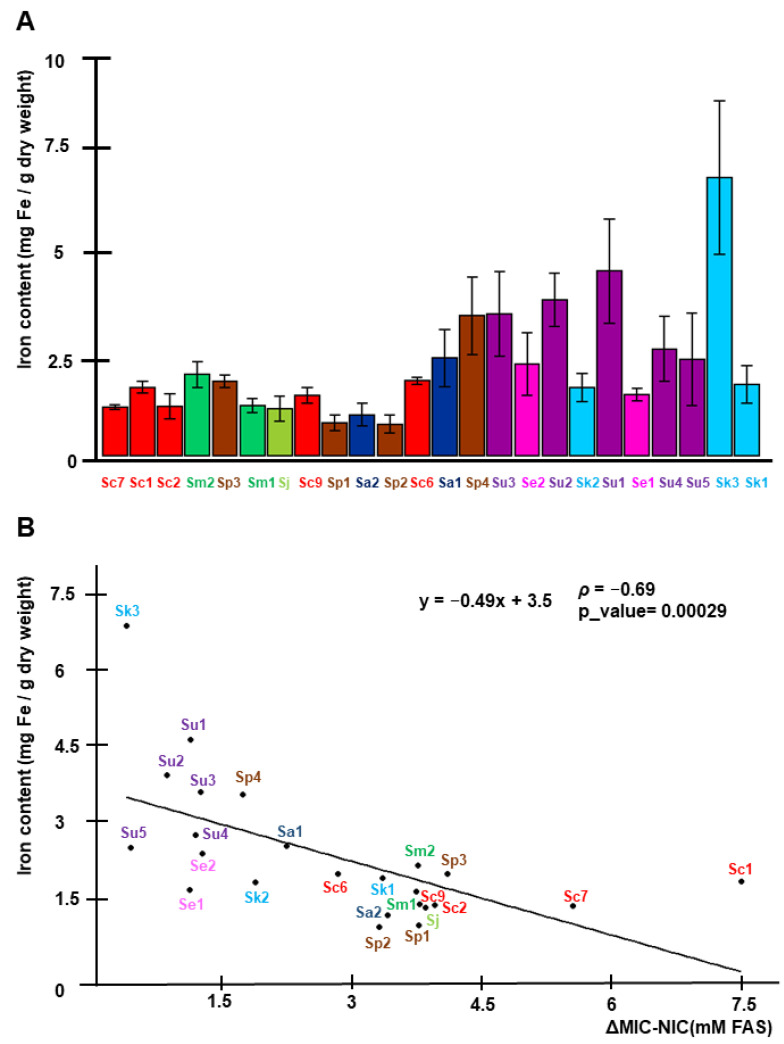
Iron accumulation under high-iron conditions and correlation with iron sensitivity. (**A**). Overnight precultures of yeast strains were reinoculated at an OD600 of 0.2 in liquid SC medium with 4 mM FAS and grown for 24 h. Then, cells were collected and endogenous iron (mg Fe/g dry weight) was determined as indicated in Materials and Methods. Data and error bars represent the average and standard deviation of at least three independent biological replicates. Strains are ordered according to the MIC values depicted in Figure 2. (**B**). Scatter plot analysis of ΔMIC-NIC (x axis) and iron content (y axis) variables. Spearman’s correlation equation is shown, together with Spearman’s rank coefficient (ρ) and a *p*_value. Colors and strain abbreviations follow the same code as in Figure 1.

In a previous characterization of various iron-resistant and iron-sensitive *S. cerevisiae* strains, we observed that the resistance to high iron was achieved by limiting the incorporation of the metal into cells, while iron-sensitive strains accumulated higher intracellular amounts of iron [13]. To determine whether this mechanism was extensive to other species of the *Saccharomyces* genus, we measured the endogenous iron levels after 24 h of incubation in SC with 4 mM FAS at 20 °C. Indeed, we observed that iron-resistant strains, like Sc7, Sm2, Sp3, Sm1, and Sj, limit the accumulation of intracellular iron (<2 mg Fe/g DW). However, iron-sensitive strains could be separated into two groups: (i) Strains that accumulate high endogenous iron levels (3–7 mg Fe/g DW), such as Sp4, Su1–3 and Sk3, and (ii) strains that are sensitive to much lower amounts of intracellular iron (around 2 mg Fe/g DW), like Sk1–2 and Se1–2 (Figure 3A). According to these observations, the Spearman correlation coefficient (ρ) between iron content and ΔMIC-NIC has a value of −0.69, suggesting a fairly strong relationship, which is statistically significant (Figure 3B). Taken together, these results suggest that iron sensitivity in the *Saccharomyces* genus could be explained by either an excessive iron accumulation, following the same tendency observed for *S. cerevisiae* strains [13], or by an increased sensitivity to intracellular iron.

### 2.2. Iron-Sensitive Strains Show Alterations in Cellular Redox State which Are Not Due to a Diminished Response to Oxidative Stress

**Figure 4:**
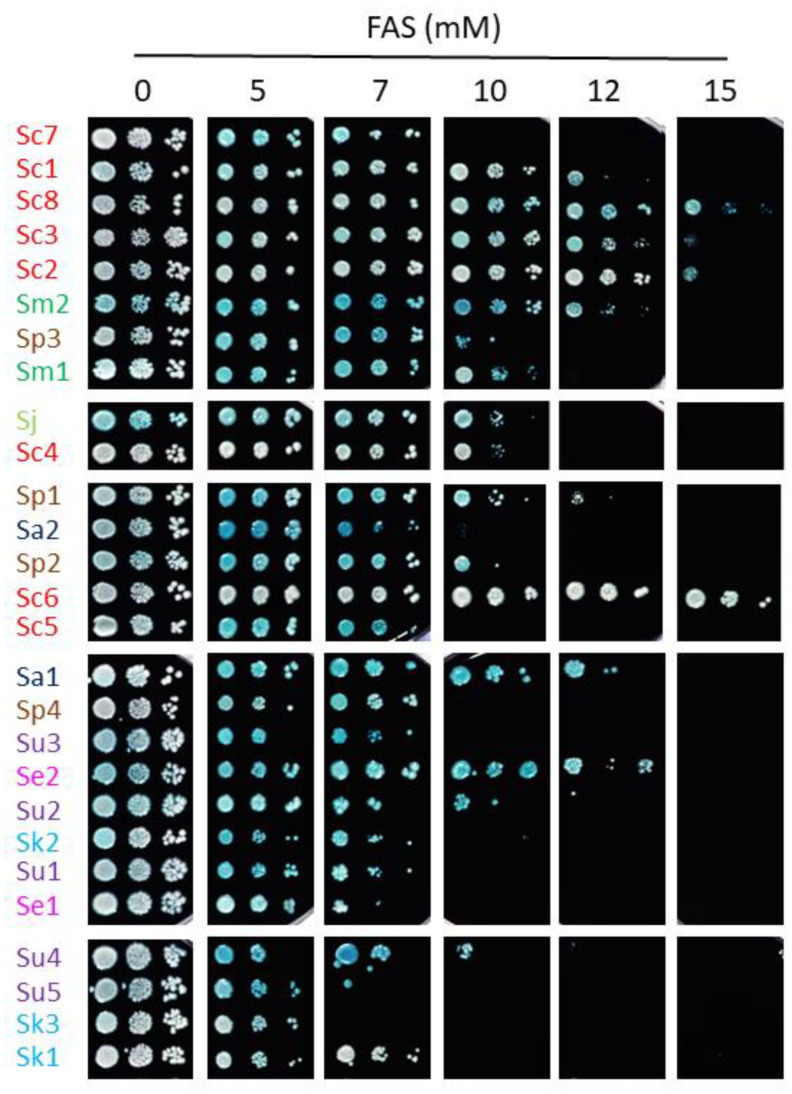
Growth of *Saccharomyces* strains in high-iron conditions. Overnight precultures were diluted to an OD_600_ of 0.1, 0.01 and 0.001 and spotted on SC + 1 mM methylene blue agar plates with the indicated range of FAS concentrations. Plates were incubated for 7 days at 20 °C and then photographed. An intense blue color indicates a more oxidized state of the cells. Strains are sorted based on MIC values and species-specific colors are used as in the above figures.

To further analyze how iron impacts the growth of different species of the *Saccharomyces* genus, we grew yeast cells in solid media containing increasing iron concentrations. For most strains, iron toxicity began to manifest at 5 mM FAS. To exclude any effects of HCl, which was used to prepare FAS stock, control plates were prepared with equivalent concentrations of HCl (Supplementary Figure S2). Particular strains displayed a growth defect for HCl at higher concentrations than for iron, indicating that iron effects were specific. While some exceptions are observed (e.g., Sc7 and Sc1 strains are more sensitive to iron in solid than in liquid media, and Sa1, Se2, and Sc6 strains grow better in solid media than expected by their liquid MIC values), strains with low MIC values in liquid media showed impaired growth when high-iron concentrations were present in solid conditions, whereas strains with higher liquid MIC values also grew better in plates (Figure 2 and Figure 4). Therefore, solid assays support the general conclusions made from liquid growth curves.

**Figure 5:**
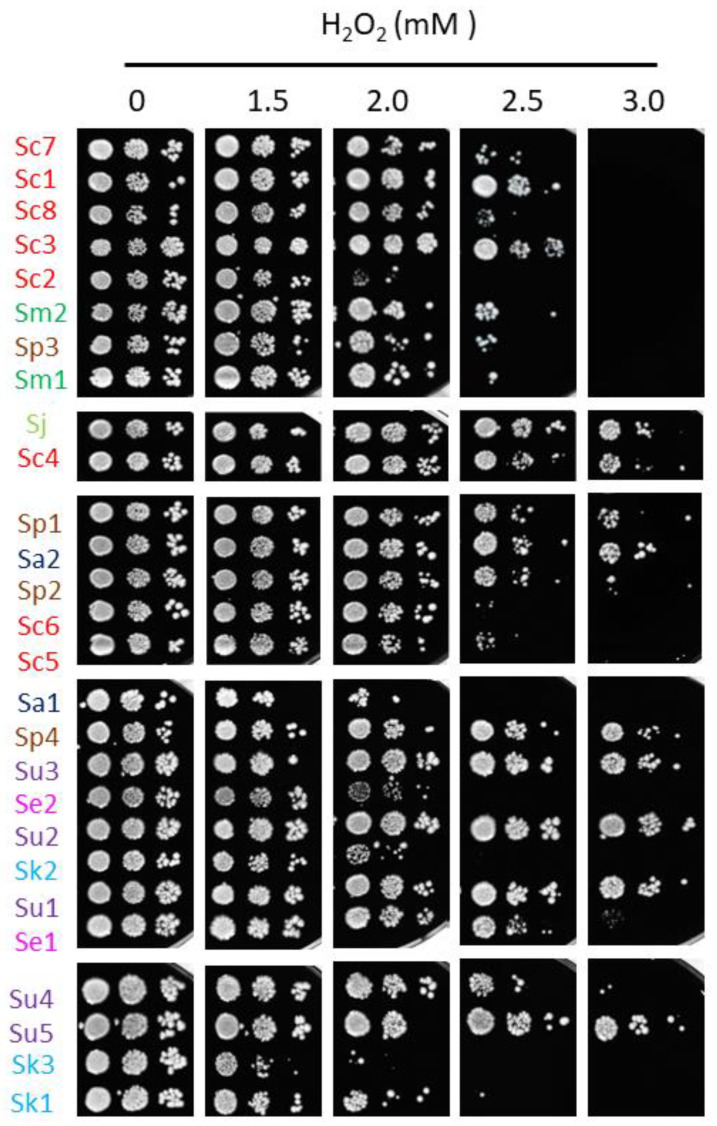
Growth of *Saccharomyces* strains in H_2_O_2_-containing media. Overnight precultures were diluted to an OD_600_ of 0.1, 0.01 and 0.001, and spotted in SC plates with a range of H_2_O_2_ concentrations. Plates were incubated during 7 days at 20 °C and then photographed. Strains are sorted based on MIC values and species-specific colors are used as in the above figures.

**Figure 6:**
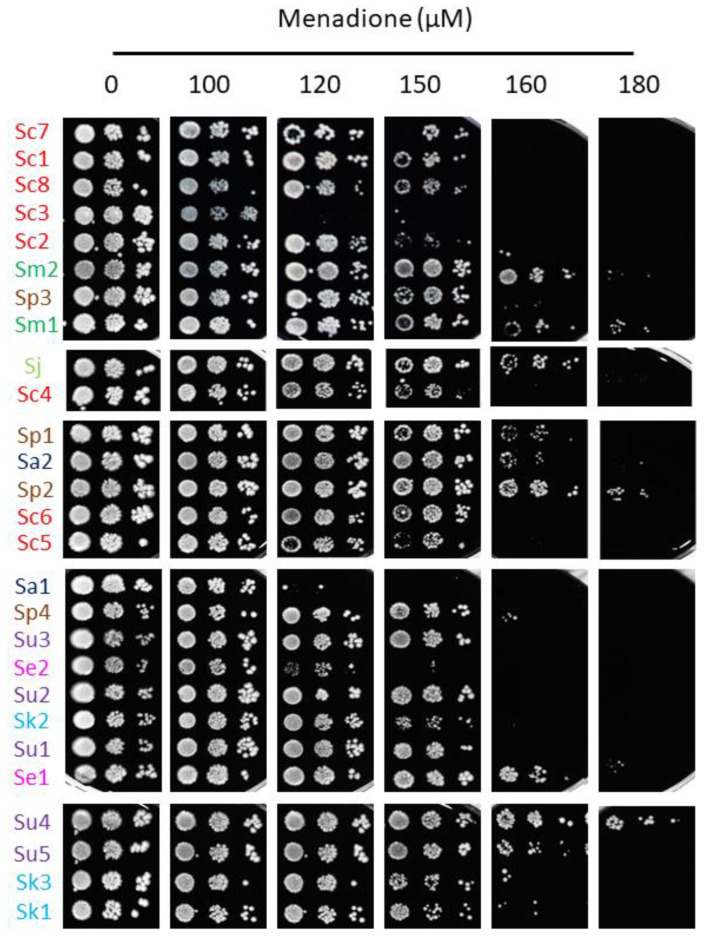
Growth of *Saccharomyces* strains in the menadione-containing media. Yeasts were cultivated and assayed for growth, as depicted in Figure 5, but on SC plates with the indicated range of menadione concentrations.

Iron toxicity has been attributed to the ability of this metal to participate in Fenton reactions, leading to ROS production which can cause oxidative damage to cellular components [7]. To ascertain whether the differences in the response to iron excess observed among the strains of this study could be explained by alterations in their cellular redox state, solid medium was supplemented with the biocompatible redox indicator methylene blue, which exhibits an intense blue color in its oxidized form and is colorless when reduced. Thus, blue yeast colonies are associated with a more oxidative cellular state [13,21]. All *S. cerevisiae* strains, except for Sc5, exhibited white or light blue color independently of iron concentration, suggesting that, besides being more resistant to iron, the change in their oxidative state is minimal. On the contrary, most non-*cerevisiae* strains, with the exception of Su2, Se1 and Sk1, showed a darker blue color after iron exposure, indicating that exposure to this metal provokes a more oxidized state. Since these observations were compatible with a general adaptation to oxidative stress, we decided to challenge these strains with two different ROS sources, H_2_O_2_ and menadione (Figure 5 and Figure 6). We observed that, differently from iron resistance, non-*cerevisiae* species, such as *S. jurei*, *S. paradoxus* (Sp1–2 and 4), *S. arboricola* (Sa1) and *S. uvarum* (Su1–3, 5), were found between the most H_2_O_2_ resistant strains (Figure 5). Strikingly, *S. uvarum* strains displayed an inverse correlation between both responses, being resistant to H_2_O_2_, but sensitive to iron. Regarding *S. cerevisiae*, only Sc4 was among the most resistant strains, while the rest of *S. cerevisiae* strains, except Sc2, which was highly sensitive, form an intermediate resistant group, together with the evolutionarily closely related *S. mikatae* strains and Sp3 (Figure 5). With respect to menadione, which causes oxidative stress through the production of superoxide anions, a slightly different patron is observed, with some exchanges between the high and medium resistant groups, but still *S. cerevisiae* strains were found in the intermediate group, overcome in resistance by *S. mikatae*, Sp1–2, Se1 and Su4–5 (Figure 6). Finally, the most H_2_O_2_-sensitive strains were also sensitive to menadione, like Sc3, Sa1, Sc2, and Se2. Taken together, these results indicate that *Saccharomyces* iron sensitivity cannot be exclusively attributed to a general decrease in oxidative stress resistance.

### 2.3. Iron-Sensitive Strains Are Less Thermotolerant

**Figure 7:**
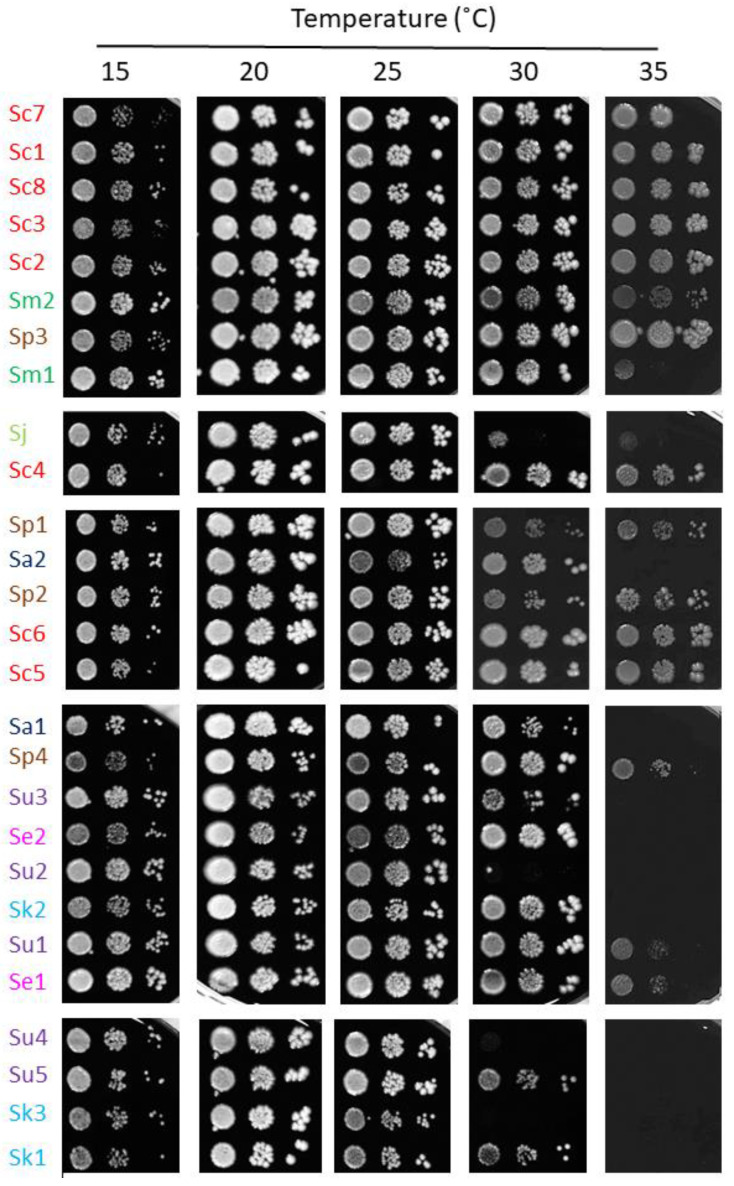
Growth of *Saccharomyces* strains at different temperatures. Yeasts were cultivated and assayed for growth, as indicated in Figure 5, but plates were incubated at 15, 20, 25, 30 and 35 °C.

The *Saccharomyces* genus contains thermotolerant (*S. cerevisiae* and *S. paradoxus*) and cryotolerant (*S. arboricola*, *S. eubayanus* and *S. uvarum*) species. To explore a potential correlation between adaptation to high temperatures and the resistance to high iron and/or oxidative stress, we studied the growth of this particular set of *Saccharomyces* strains at different temperatures, ranging from 15 °C to 35 °C (Figure 7). First, we confirmed that, as reported in References [17,20], *S. eubayanus* and *S. uvarum* are thermosensitive species, where growth at 30 °C is compromised. The *S. jurei* strains used here, *S. arboricola* and *S. kudriavzevii*, also performed better at low temperatures and were unable to properly grow at 35 °C (Figure 7). However, *S. cerevisiae* and its closest relative, *S. paradoxus*, are the most thermoresistant, growing relatively well at 35 °C, but also the most cryosensitive, with growth defects at 15 °C. The remainder of the species showed an intermediate response, with variations among strains. These results show a putative link between iron resistance and thermotolerance adaptations.

### 2.4. Iron-Sensitive Strains Show Altered Expression of Iron Homeostasis and Oxidative Response Genes

**Figure 8:**
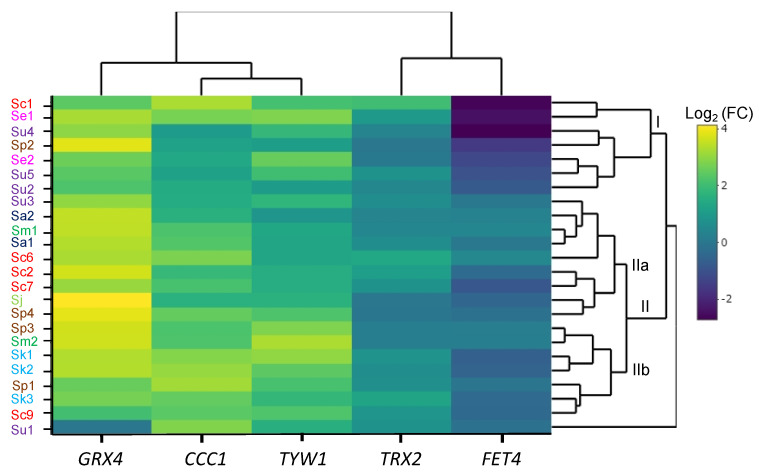
Effect of iron on gene expression in *Saccharomyces* strains. Yeast strains were exposed to 2 mM FAS for 1 h, as described in the Materials and Methods, and *GRX4*, *CCC1*, *TYW1*, *TRX2* and *FET4* mRNA levels were determined by RT-qPCR. The heatmap represents the log_2_ fold change between SC + 2 mM FAS and SC mRNA levels, with a hierarchical cluster calculated by the Euclidean distance. Higher fold changes are represented with yellow to light green and low fold changes are depicted with light to dark blue. mRNA levels were normalized using the *ACT1* gene. Groups I, IIa and IIb are indicated. All values are the average of three independent biological replicates.

In order to decipher whether genes that are regulated in response to excess iron or oxidative stress are dysregulated in the iron-sensitive strains, we grew yeast cells to the exponential phase in SC with or without 2 mM FAS and used RT-qPCR to analyze the expression of *CCC1*, *GRX4*, *TYW1*, *FET4*, and *TRX2* genes in the different species (Table 2 and Table S2, Figures S3–S6). A hierarchical cluster heatmap was created from the log_2_ fold change of mRNA levels between iron addition and the control (SC) (Figure 8). We observed that, upon iron addition, the upregulation of Yap5 targets, *CCC1*, *GRX4* and *TYW1*, previously reported for *S. cerevisiae* [11,12,22], was conserved in all the species of the *Saccharomyces* genus (Figure S4). *GRX4* exhibited a higher increase in expression, followed by *CCC1* and *TYW1. TRX2* levels remained stable or lightly increased, whereas *FET4* expression decreased or was not altered, depending on the specific strain (Figure S5). Hierarchical clustering of expression grouped *Saccharomyces* strains in the two main groups (Figure 8), with *S. uvarum* Su1 remaining unclustered. Remarkably, six out of seven components of group I (Sc1, Se1, Su4, Se2, Su5, and Su2) were iron-sensitive strains that showed the largest shutdown of *FET4* expression and high *GRX4* mRNA levels in normal conditions (Figures S4 and S5). However, the largest group II of strains showed a milder downregulation of *FET4* and a larger induction of *GRX4* and *CCC1*. This cluster can be divided into two different subgroups, IIa and IIb. Subgroup IIa is formed by a mixture of sensitive and resistant strains (Su3, Sa2, Sm1, Sa1, Sc6, Sc2, Sc7, Sj, and Sp4). This stands out as the highest increase in *GRX4* mRNA levels, which can be attributed to the very low expression observed without iron addition, but a low increase in *TYW1* mRNA levels and an intermediate induction of *CCC1* (Figure 8 and Figure S4). Subgroup IIb is composed of the resistant strains Sp3 and Sm2, the sensitive strains Sp1, Su1, Sk1–3, and the laboratory strain Sc9. The increase in *GRX4* expression is still high in these strains, and together with Sc1, Sc6 and Se1 strains, this group shows the highest induction of *CCC1* and *TYW1*. Interestingly, the most relevant downregulation of *FET4* is observed in the *S. kudriavzevii* strains. Finally, Su1 shows a divergent pattern from all other strains, mainly marked by the downregulation of *GRX4*. Regarding *TRX2* expression, as representative of the oxidative stress response, we observed only minor increases in the presence of iron, which are only remarkable in *S. cerevisiae* and *S. kudriavzevii* strains. Conversely, *TRX2* mRNA levels remain quite unchanged upon iron addition in *S. mikatae* and *S. paradoxus* strains, or even show a slight decrease in *S. jurei* or Sp4. Other genes involved in oxidative stress, such as *YAP1*, *SOD1* and *TSA1*, were also analyzed, but no remarkable changes were observed upon iron addition (Figure S6). Taken together, these results indicate that the transcriptional response to iron excess is predominant over that of oxidative stress. While strain-specific patterns were observed, several iron-sensitive strains clustered in group I displayed a strong downregulation of the *FET4* iron transporter gene.

### 2.5. Iron-Sensitive Strains Have Impairments on Iron Toxicity Response

**Figure 9:**
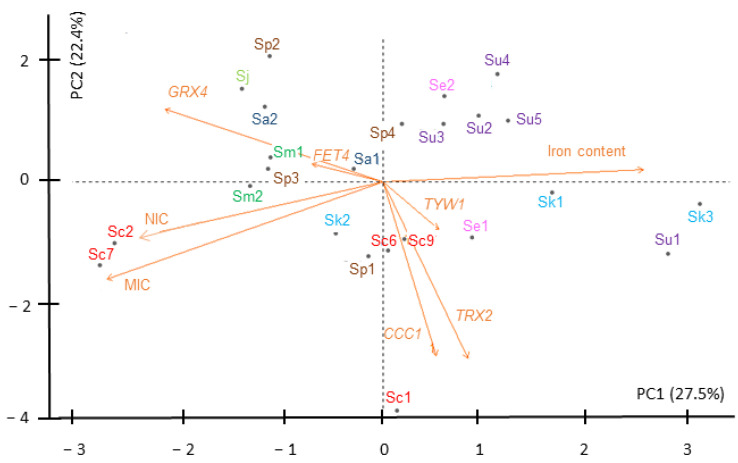
Behavior of *Saccharomyces* strains according to iron sensitivity, iron accumulation and gene expression. Two-dimensional principal component analysis (PCA) biplot of NIC, MIC, iron accumulation and gene expression of *Saccharomyces* strains under high-iron exposure. Contribution of each variable to the variance is represented by the arrow length of the vectors. Strains (individuals) are depicted by the color code and abbreviations used in previous figures.

To further dissect how the different variables (NIC, MIC, iron content and expression) influenced the iron performance of the *Saccharomyces* strains, we analyzed the above data (summarized in Table S3) by a principal component analysis (PCA) (Figure 9). We identified two principal components that described 50% of the total variance. The first principal component, PC1, which explains 27.5% of the variance, shows a high positive contribution of iron content and *TRX2* expression, and a high negative component loading of NIC, MIC, *GRX4* fold-change and, in a minor way, *FET4* expression. PC2 is responsible for 22.4% of variance, and is highly contributed by GRX4 fold changes, and shows a high negative component loading of changes in the expression of *CCC1*, *TRX2*, and, to a lesser extent, *TYW1*, as well as NIC and MIC. Iron content has little contribution to this component. Additionally, PC3 and PC4 represent the 15.9% and 13.9% of the variance, respectively (Figure S7). However, changes in expression of *TYW1* and *CCC1* highly correlate between them, as expected for Yap5 targets, and also with changes in *TRX2*, but correlate inversely with *GRX4*, the other Yap5 target, and with *FET4* variations. *GRX4* and *FET4* mRNA changes, on the contrary, show a positive correspondence with high NIC and MIC values and inversely correlate to iron content. However, iron content only shows a small positive correlation with changes in expression of *TYW1*, but not of other genes. Strains are dispersed around the four quadrants forming small groups according to the influence of the different variables on their behavior upon iron addition. On the left, there are strains with relatively high NIC and MIC values, largely influenced by changes in *GRX4* mRNA levels. An iron-resistant group composed of Sm1, Sm2, Sp2, Sp3, Sj, and Sa2 show a negative correlation with changes in *CCC1*, *TYW1* and *TRX2*. On the right side, there are strains with low NIC and MIC values. Sc1, Se1, Sc6, Sc9 and crossing the y axis, Sp1 also present an iron content similar to the resistant strains, high positive correlation with *TYW1*, *CCC1* and *TRX2* changes and a high negative correlation with changes in *GRX4* and *FET4* expression, being Sc1 an outlier. At some distance in this quadrant we found Sk3 and Su1, which are the strains with the highest iron content. They also show a low contribution of changes in gene expression. Finally, there is a group formed by four *S. uvarum* strains, Su2–5, grouped in the above right quadrant with Se2, and close to the axis, to Sp4. They show an elevated iron content and low NIC and MIC values. In summary, PCA supports that: (i) Iron-resistant strains (high MIC values) have low iron contents, an elevated correlation with *GRX4* fold changes, and variations in *FET4* expression; (ii) iron-sensitive strains with low iron contents are more influenced by *CCC1*, *TYW1* and *TRX2* expression changes and by a downregulation of *FET4* expression; and (iii) iron-sensitive strains with high-iron contents are less influenced by changes in *CCC1*, *TYW1* and *TRX2* expression.

## 3. Discussion

In the last years, we have attended to a huge increase in the sequenced genomes available of the *Saccharomyces* genus, as well as comparative genetic and phenotypic studies, elevating this genus to a eukaryotic model system to study processes of adaptation and evolution [17,18]. In this work, we took advantage of this progress to expand our previous analyses on the response of *S. cerevisiae* strains from different origins to changing environmental iron levels to the *Saccharomyces* genus. For this purpose, we grew several strains from the different species in solid and liquid media supplemented with a wide range of iron concentrations. We observed differences between species regarding iron performance, with strains closely related to *S. cerevisiae* being more resistant to high-iron than the most distant ones, like *S. kudriavzevii*, *S. uvarum*, and *S. eubayanus*. Sc3, Sc7, and Sc8 were the most iron resistant strains, probably because they have not been isolated from wild niches, but instead are of human origin or domesticated lineages. Conversely, the rest of the strains were isolated from soils, decayed leaves, or barks from Fagales order, which are probably niches with low bioavailable iron content (Table S1 [17,19]). As shown for other conditions, *S. mikatae* and *S. jurei* behaved similarly in response to iron excess, as well as *S. uvarum* and *S. eubayanus*, probably because they are the less divergent sister-species in the genus [17]. *S. cerevisiae* and *S. paradoxus* strains showed a more diverse phenotype, including iron-sensitive and iron-resistant varieties.

We also determined the iron content of all strains after exposure to high-iron levels. Two different behaviors were observed in the sensitive strains: (i) Strains that acquired high levels of iron from the medium and (ii) strains that were highly sensitive to low intracellular iron content. Even so, there is a quite strong inverse correlation between iron content and ΔMIC-NIC. Given that yeasts do not possess a regulated iron excretion mechanism, a higher iron accumulation indicates that cells are not able to limit iron entrance when the metal is in excess (as reviewed in Reference [23]). This consideration would be in agreement with our previous observations for the *S. cerevisiae* strains, which showed that iron-sensitive strains accumulate high intracellular iron levels, whereas iron-resistant strains had to be exposed to higher extracellular iron concentrations to become iron overloaded [13]. Moreover, Lindahl’s group has demonstrated that the lack of synchronization between iron uptake and cell growth leads to iron overload [24]. As shown in Table S4, this seems to be the case for most iron-sensitive strains analyzed in this study, which only duplicate once or twice when cultivated for 24 h at 4 mM iron. Similarly, another study has shown that *S. cerevisiae* flor strains from the Magarach collection, which are iron-sensitive, possess a codon stop in the *AFT1* gene that leads to a truncated Aft1 protein, which lacks the last 42 amino acids [15]. The loss of Aft1 carboxy-terminal region, which may be involved in post-translational regulation of this transcriptional factor, leads to an increase in iron uptake [15]. Siderophore-bound iron uptake is also important for adaptation to iron limitation, even for yeast species that lost the ability to synthetize these compounds, such as *S. cerevisiae*, and plays an ecological role in the competition with other microorganisms [22]. Thus, particular species of budding yeasts have adapted to iron scarcity through the reacquisition of this lost ability by horizontal operon transfer from bacteria [22]. Therefore, increasing iron import efficiency from the environment seems to be a common strategy to overcome iron deficiency. On the other hand, we also found a group of strains that are highly sensitive to iron but accumulate low to moderate iron levels. It has been proposed that the two main strategies to cope with metal stress are “avoidance” or “sequestration” [25]. Therefore, these strains may be able to shut down iron import, in a similar manner than resistant strains, but they would be unable to detoxify iron properly once it is acquired from the medium, possibly because of defects in their sequestration machineries. Thus, Sc1 and Sc4 strains accumulate iron levels similar to the laboratory strain BY4741 at 30 °C, despite they are highly iron-sensitive [13] and, particularly, Sc1 presents loss of function mutations in *CCC1* and *YAP5* genes, which may be the molecular cause of its behavior [14]. Independently of the reason behind, we observed that iron-sensitive *Saccharomyces* strains are generally more oxidized than resistant strains at the same iron concentrations, in agreement with other studies [10,15,18]. Thus, iron toxicity could reside in cytosolic accumulation due to ineffective sequestration, which could lead to increased oxidative damage. Supporting this idea, overexpression of vacuolar or mitochondrial transporters has been shown to compensate the iron toxicity observed in *ccc1*Δ mutants [26,27].

To further analyze the involvement of oxidative and/or other stress responses in iron performance, we exposed strains to different oxidative sources and variations in temperature. While we could not establish a strong correlation between high-iron resistance and any other condition tested, the most iron-sensitive strains seemed to be those that performed well at low temperatures, like *S. uvarum*, *S. eubayanus*, and *S. kudriavzevii*. Further analyses will be necessary to determine a possible correlation between these two phenotypes. Finally, we also determined the effect of iron addition on the expression of genes involved in iron homeostasis and oxidative response. Generally, expression of the iron-responsive genes *CCC1*, *TYW1*, and *GRX4*, as well as the oxidative stress gene *TRX2* increased when iron was added. We observed a different expression pattern in a group of iron-sensitive strains, whereas other sensitive groups behaved more similarly to the iron-resistant strains. Thus, some iron-sensitive strains, such as Su2–5, Sp2 and Sa2, exhibit the lowest fold changes for *CCC1*, and in most cases, *TYW1* expression, which could match with an impaired iron sequestration or detoxification performance, as discussed above. Instead, other sensitive strains, like Sk1–2, show expression changes similar to resistant strains, such as Sm1–2 or Sp3, with moderate increases in *CCC1* as well as in *TYW1* and *GRX4* levels, and a considerable decrease in *FET4* expression, which could account for the similar intracellular iron content in these strains. These results also suggest that an insufficient upregulation of *CCC1* could lead to a rise in iron that could be compensated by *TYW1* and *GRX4*, which are shown to be involved in iron-buffering [11]. Interestingly, some iron-sensitive strains, such as Sp2, Se1–2, and Su4 have high *FET4* mRNA levels in normal conditions, similar to the Malaysian strain Sc1. Thus, these strains seem to detect iron deficiency in normal conditions as if the threshold for iron detection was shifted towards smaller concentrations of the metal [14]. A high expression in oxidative resistance genes has been linked to adaption to iron resistance; however, the only oxidative stress gene that presents a significant change is *TRX2*; this rise has been described as being especially important in the *S. cerevisiae* strains Sc1, Sc2, and Sc7 [16]. Moreover, it has been shown that iron toxicity is still present in anaerobiosis conditions and the overexpression or deletion of oxidative stress genes does not rescue *ccc1*Δ sensitivity to iron [10]. Therefore, it is possible that other factors, such as impaired sphingolipid signaling or iron mismetallation, could also be influencing the response to iron of the yeast strains analyzed in the present work [8,9]. Further experiments should be done to decipher additional mechanisms that enable Saccharomyces species to survive in environments with a high-iron content.

## 4. Materials and Methods

### 4.1. Yeast Strains and Growth Conditions

All the *Saccharomyces* species strains used in this study are listed in Table 1. Yeast strains were stored at −80 °C in cryotubes in yeast extract-peptone-dextrose (YPD) medium and 15% glycerol. Routine cultures were maintained in YPD plus 2% agar plates at 20 °C. Unless otherwise indicated, yeasts were grown in synthetic complete (SC) medium at 20 °C to allow for a similar comparative growth of all species [17]. Iron was added at the indicated concentrations from a 100 mM ferrous ammonium sulfate (FAS, Merck-Sigma, Darmstadt, Germany) stock solution, prepared freshly in 0.1 M HCl to promote iron solubility. A control with the equivalent HCl concentration was prepared for each experiment. The redox indicator methylene blue (Merck-Sigma, Darmstadt, Germany) was added to agar plates with FAS or HCl to a 1 mM final concentration. For growth spot assays, overnight precultures were adjusted to an OD_600_ of 0.1, 0.01, and 0.001, and then spotted on SC agar plates with the indicated concentrations of FAS, H_2_O_2_ (Honeywell, Charlotte, NC, USA), menadione (Merck-Sigma, Darmstadt, Germany) or HCl. Plates were incubated for 7 days at 20 °C and then photographed. However, temperature spot assays were performed at 15, 20, 25, 30, and 35 °C.

### 4.2. Determination of Non-Inhibitory and Minimal Inhibitory Concentrations

Yeast strains were inoculated in triplicate in 96-well plates at an OD_600_ of 0.1 in SC liquid media and growth at 20 °C was registered measuring OD_600_ every 30 min with an absorbance microplate reader with a stacker microplate handling system, Spectrostar Omega (BMG Labtech, Ortenberg, Germany). The area under the curve (AUC) was obtained using Growth Curve Analysis Tool (GCAT) online software [28]. This value was used to calculate the fractional area (Fa = AUC_Fe_/AUC). Fa was plotted versus log_10_ of FAS concentration. Non-inhibitory (NIC) and minimal inhibitory concentration (MIC) values were calculated, as previously described [29], using an in-house script in R Studio 2022.07.1, version 4.2.0 [30], with “nlsLM” tool from minpack.lm package 1.2–2 [31]. Segment representation was obtained using the “geom_segment” tool of ggplot2 package 3.3.3 [32].

### 4.3. Endogenous Iron Measurements

To determine endogenous iron levels, a previously described colorimetric assay was used [33]. First, cells were inoculated at 0.2 OD_600_ in SC medium supplemented with 4 mM FAS for 24 h. Then, 1 mL of cells at 5 OD_600_ units /mL, or an equivalent amount of cells, were harvested and washed twice with 1 mM EDTA and once with milliQ water. Cell pellets were dissolved and digested in 3% HNO_3_ at 98 °C for at least 16 h. After digestion, ascorbic acid and ammonium acetate (Merck-Sigma, Darmstadt, Germany) were added to cell extracts to a final concentration of 1 M and 40 mM, respectively. Finally, 3.2 mM of the Fe^2+^-specific chelator bathophenanthroline disulfonic acid disodium, BPS (Merck-Sigma, Darmstadt, Germany) was added and absorbance at 535 nm (A_535_) was measured. For dry weight determination, 6 mL of cells grown in the same conditions described above were collected at 5 OD_600_ units/mL, dried at 50 °C for 3 days, and weighed. Spearman correlation coefficient between iron content and ΔMIC-NIC was obtained with “ggplot2” R Studio 2022.04.22 [30], 4.2.0 package.

### 4.4. RNA Processing and Analysis

Overnight precultured cells were inoculated at an OD_600_ of 0.2 in SC liquid medium during 5 h in glass flasks. Afterwards cells were transferred to fresh medium without or with 2 mM FAS for an extra hour. Then, cells were harvested by centrifugation and frozen. Total RNA isolation and particular mRNA levels were determined by RT-qPCR, as previously described [34]. Specific primers used for RT-qPCR are listed in Table S2. The *ACT1* mRNA values were used to normalize.

Genome assemblies. Previously published genome assemblies were downloaded from NCBI and ATCC web portal. Accession numbers or links are provided in Table S1.

### 4.5. Phylogenetic Tree

A Neighbor-Joining (NJ) tree was generated based on the fast method fastANI 1.1 [35]. Then, we calculated the pairwise average nucleotide identity (ANI) among genome assemblies, with a fragment length set to 400 bp. FastANI values were then converted to a percentage dissimilarity matrix by subtracting ANI from a value 100%. All calculations and matrix formatting were done in RStudio 2022.07.1, R 4.0.2 [30], using dplyr 1.0.5, stringr 1.5.3 and reshape2 1.4.4 packages. The dissimilarity data was used as the distance to reconstruct the NJ phylogenetic tree in MEGA v5 [36]. The phylogenetic tree in newick format was rooted to the *S. eubayanus* and *S. uvarum* branch in the R using ape 5.4 [37], phytools 0.7 [38] and the phylogenetic tree was drawn with ggtree 2.2.4 [39].

### 4.6. Gene Expression Heatmap

The heatmap representation of log_2_ fold change was obtained with the “heatmaply” tool of heatmaply 1.3.0 package [40] from R Studio 2022.04.22 4.2.0 [30].

### 4.7. Principal Component Analyses

The principal component analysis (PCA) of the variables was obtained in R Studio 2022.04.22 [30], 4.2.0 using the “prcomp” tool of factoextra 1.0.7 package [41]. The “fviz_pca_biplot” tool from ggfortify 0.4.14 package [42] was used to represent the two-dimensional PCA biplot of variables and individual strains.

## 5. Conclusions

Iron in excess is toxic for eukaryotic cells. The budding yeast *S. cerevisiae* mainly responds to excessive iron in the environment by restricting its uptake, as iron-resistant strains usually accumulate less intracellular iron than iron-sensitive ones [13]. In this work, we analyzed the response of diverse species of the *Saccharomyces* genus to elevated iron levels, and found that *S. cerevisiae* and its closer species in evolution (*S. paradoxus*, *S. jurei* and *S. mikatae*) generally show a higher resistance to iron than more distant species, such as *S. eubayanus* and *S. uvarum*, with *S. kudriavzevii* being especially iron-sensitive species. Two different behaviors were observed for the iron-sensitive strains. While some strains accumulated high intracellular iron levels, other strains were able to limit its incorporation, but not to properly sequester or detoxify it. Additionally, iron-sensitive strains showed a higher oxidized state, but in general, the response to iron of the *Saccharomyces* strains did not correlate with the responses to oxidative stress or variations in temperature, indicating that it is a specific response. The altered expression of iron homeostasis genes, such as the vacuolar iron importer *CCC1* or the iron transporter *FET4*, shows a certain correlation with the iron performances of different groups of strains, while the expression of oxidative response genes, such as *TRX2*, has a lesser influence.

## Figures and Tables

**Table 1:** *Saccharomyces* strains used in this work. Species are listed in order of evolutionary proximity to *S. cerevisiae*, according to the phylogenetic tree depicted in Figure 1. * Classification according to References [17,18].

Abbreviation	Species	Strain	Geographical Origin	Population *
Sc1	*S. cerevisiae*	UWOPS03-461.4	Asia (Malaysia)	19. Malaysian
Sc2	*S. cerevisiae*	CECT10131	Europe (Spain)	8. Mixed origin
Sc3	*S. cerevisiae*	CECT10711	Asia (Japan)	25. Sake
Sc4	*S. cerevisiae*	CECT10120	Europe (Spain)	8. Mixed origin
Sc5	*S. cerevisiae*	CLIB219-2B	Europe (Russia)	18. Far East Asia
Sc6	*S. cerevisiae*	YPS128	USA (Pennsylvania)	23. North American oak
Sc7	*S. cerevisiae*	YJM978	Europe (Italy)	1. Wine/European
Sc8	*S. cerevisiae*	CECT11032	Europe (Italy)	1. Wine/European
Sc9	*S. cerevisiae*	BY4743		
Sp1	*S. paradoxus*	yHDPN24	Canada (Québec)	America C
Sp2	*S. paradoxus*	N44	Europe (Russia)	Far East
Sp3	*S. paradoxus*	YPS138	USA (Pennsylvania)	America B
Sp4	*S. paradoxus*	CBS432	Europe (Russia)	EU
Sj	*S. jurei*	NCYC3947	Europe (France)	EU
Sm1	*S. mikatae*	yHAB336	Asia (China)	Asia B
Sm2	*S. mikatae*	IFO1815	Asia (Japan)	Asia A
Sk1	*S. kudriazevii*	IFO1802	Asia (Japan)	Asia A
Sk2	*S. kudriazevii*	ZP591	Europe (Portugal)	EU
Sk3	*S. kudriazevii*	IFO1803	Asia (Japan)	Asia B
Sa1	*S. arboricola*	CBS10644	Asia (China)	Asia A
Sa2	*S. arboricola*	ZP960	Oceania (New Zealand)	Oceania
Se1	*S. eubayanus*	yHCT69	South America (Argentina)	Patagonia B/HOL
Se2	*S. eubayanus*	yHRVM107	USA (North Carolina)	Patagonia B/HOL
Su1	*S. uvarum*	yHAB60	Unknown	HOL/SA-A
Su2	*S. uvarum*	CBS7001	Europe (Spain)	HOL-EU
Su3	*S. uvarum*	yHAB521	South Ameria (Argentina)	SA-B
Su4	*S. uvarum*	ZP964	Asia (New Zealand)	Australasia
Su5	*S. uvarum*	yHCT77	USA (Oregon)	HOL-NA

**Table 2:** List of genes involved in iron overload and oxidative stress analyzed in this study. Systematic and common gene names, a brief description of function, and human orthologues are indicated.

Systematic Name	Common Name	Brief Description	Human Ortholog
YLR220W	*CCC1*	Vacuolar Fe^2+^/Mn^2+^ transporter	-
YER174C	*GRX4*	Glutathione-dependent oxidoreductase and glutathione S-transferase	*GLRX3*
YPL207W	*TYW1*	Iron-sulfur protein required for synthesis of wybutosine modified tRNA	*TYW1A* and *TYW1B*
YMR319C	*FET4*	Plasma membrane low-affinity Fe^2+^ transporter	*-*
YGR209C	*TRX2*	Cytoplasmic thioredoxin isoenzyme	*TXN*
YML007W	*YAP1*	Basic leucine zipper (bZIP) transcription factor	
YJR104C	*SOD1*	Cytosolic copper-zinc superoxide dismutase	*SOD1*
YML028W	*TSA1*	Thioredoxin peroxidase	*PRDX1*, *PRDX2*, *PRDX3* and *PRDX4*

## Data Availability

The data presented in this study are openly available in Digital CSIC (https://digital.csic.es) at https://doi.org/10.20350/digitalCSIC/14763 (accessed on 6 October 2022).

## Supplementary Materials

The following are available online at https://www.mdpi.com/article/10.3390/ijms232213965/s1.
