# Supplementary material for: Adaptation of Saccharomyces Species to High-Iron Conditions
Source: Int J Mol Sci. 2022 Nov 12;23(22):13965. doi: 10.3390/ijms232213965 (PMC9693265; doi:10.3390/ijms232213965)
Supplement: Supplementary file 1 [file ijms-23-13965-s001.zip › Supplementary Figures.pdf]

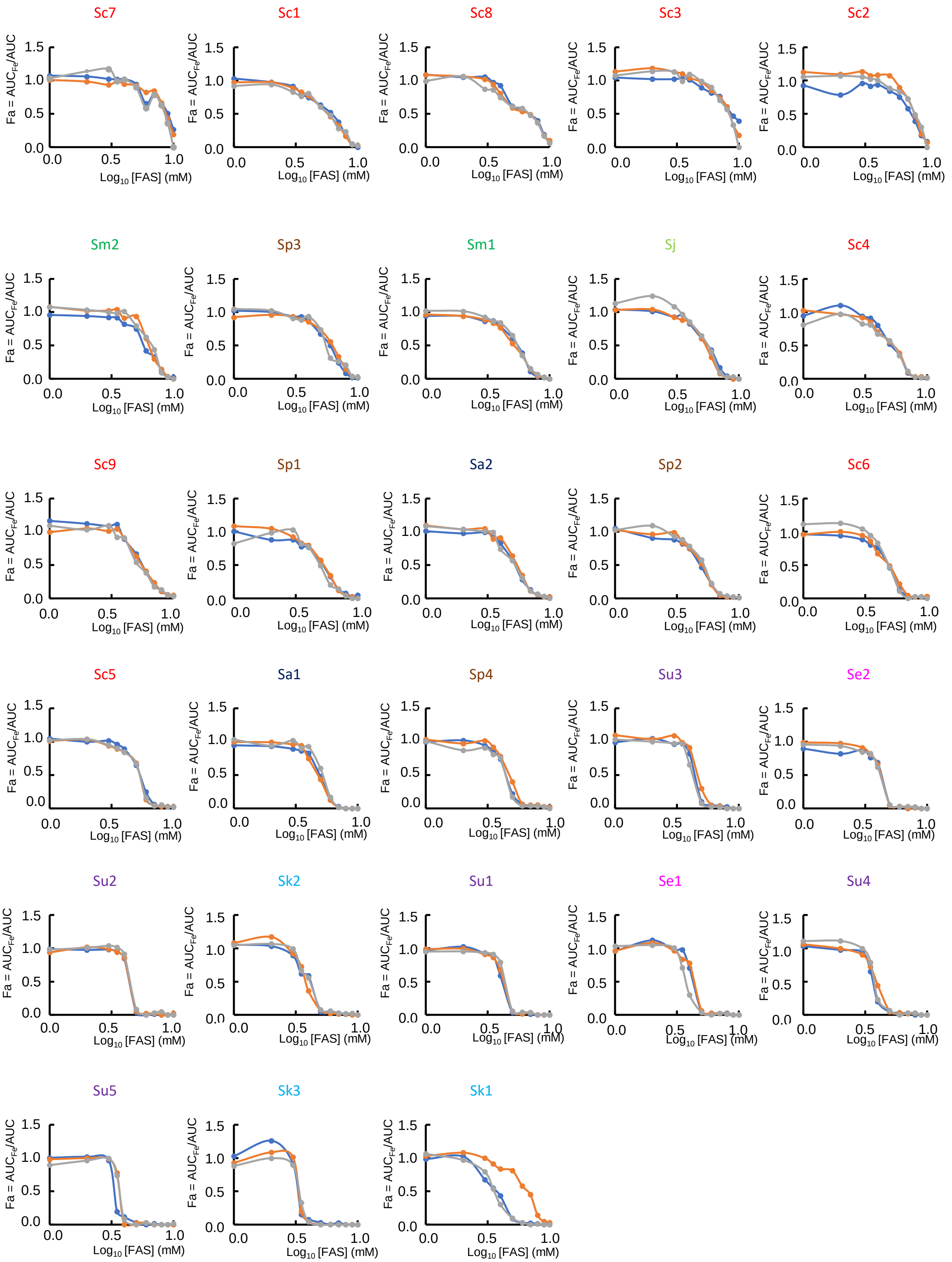


**Figure S1. Representation of the fractional area vs. FAS concentration.** Dots and lines represent the value of fractional area (Fa= $AUC_{Fe}$ / AUC) against $log_{10}$ of FAS concentration (mM) for each strain replicate. Strain names are colored as in table 1 and ordered according to MIC values in figure 2.

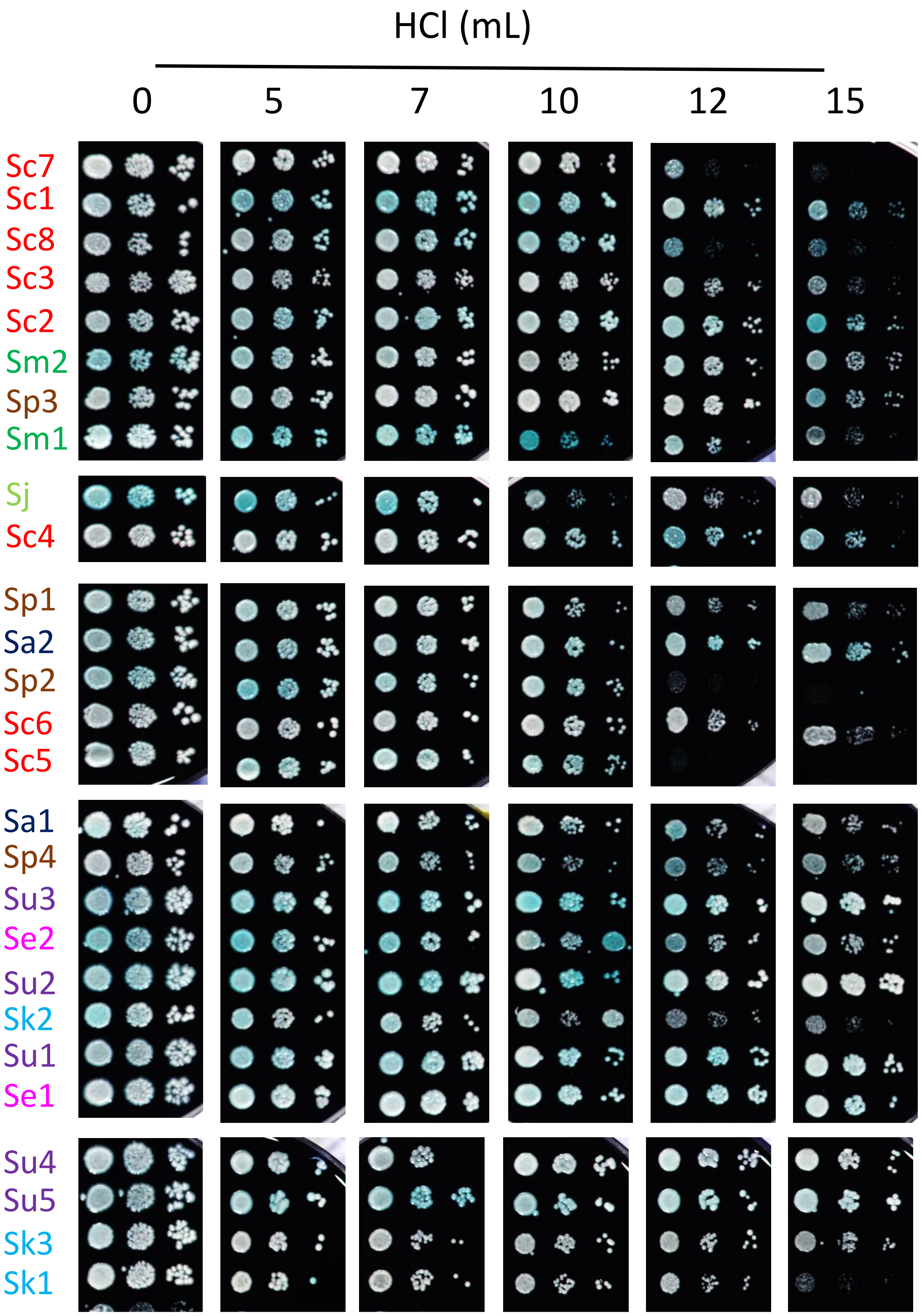


**Figure S2**. **Growth of *Saccharomyces* strains in HCl-containing media.** Overnight precultures were diluted to an $OD_{600}$ of 0.1, 0.01, and 0.001 and spotted on SC + 1 mM methylene blue agar plates with the indicated range of HCl volumes. Plates were incubated for 7 days at 20°C and then photographed. An intense blue color indicates a more oxidized state of the cells. Strains are sorted based on MIC values and species-specific colors are used as in the above figures.

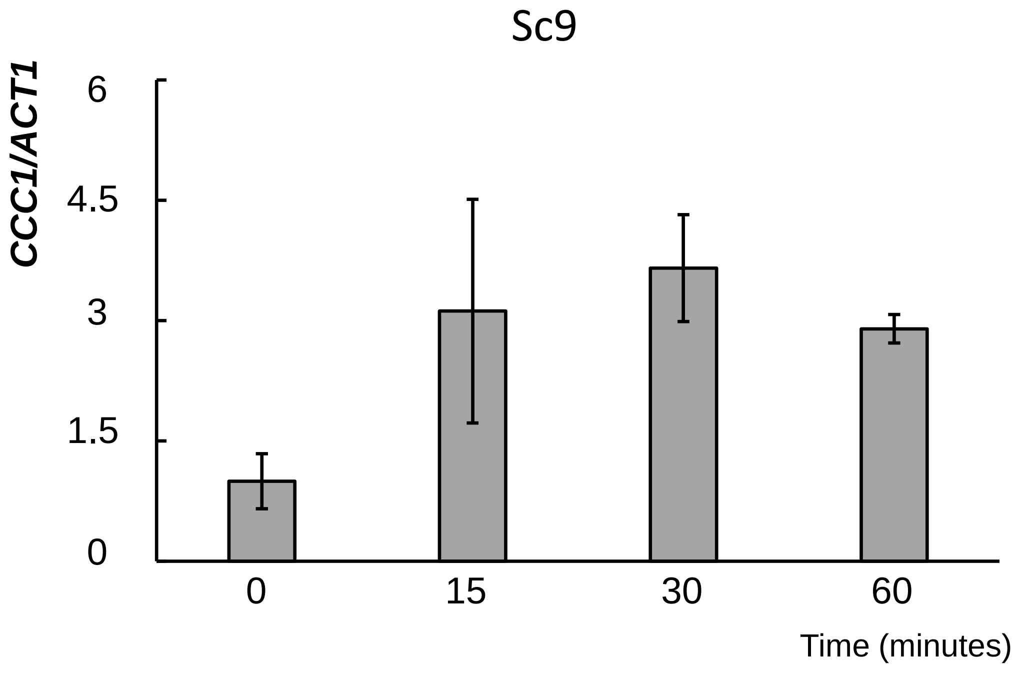


**Figure S3. Effect of iron on *CCC1* expression in Sc9 strain at different times.** Yeast strains were exposed to 2 mM FAS for 60 minutes, as described in Materials and Methods, and *CCC1 mR*NA levels were determined by RT-qPCR at 0, 15, 30 and 60 minutes. Bar plots represent the mRNA levels of *CCC1* gene at different points, relative to *ACT1* mRNA levels.Shown values are the average of 2 replicates and error bars constitute the standard deviation.

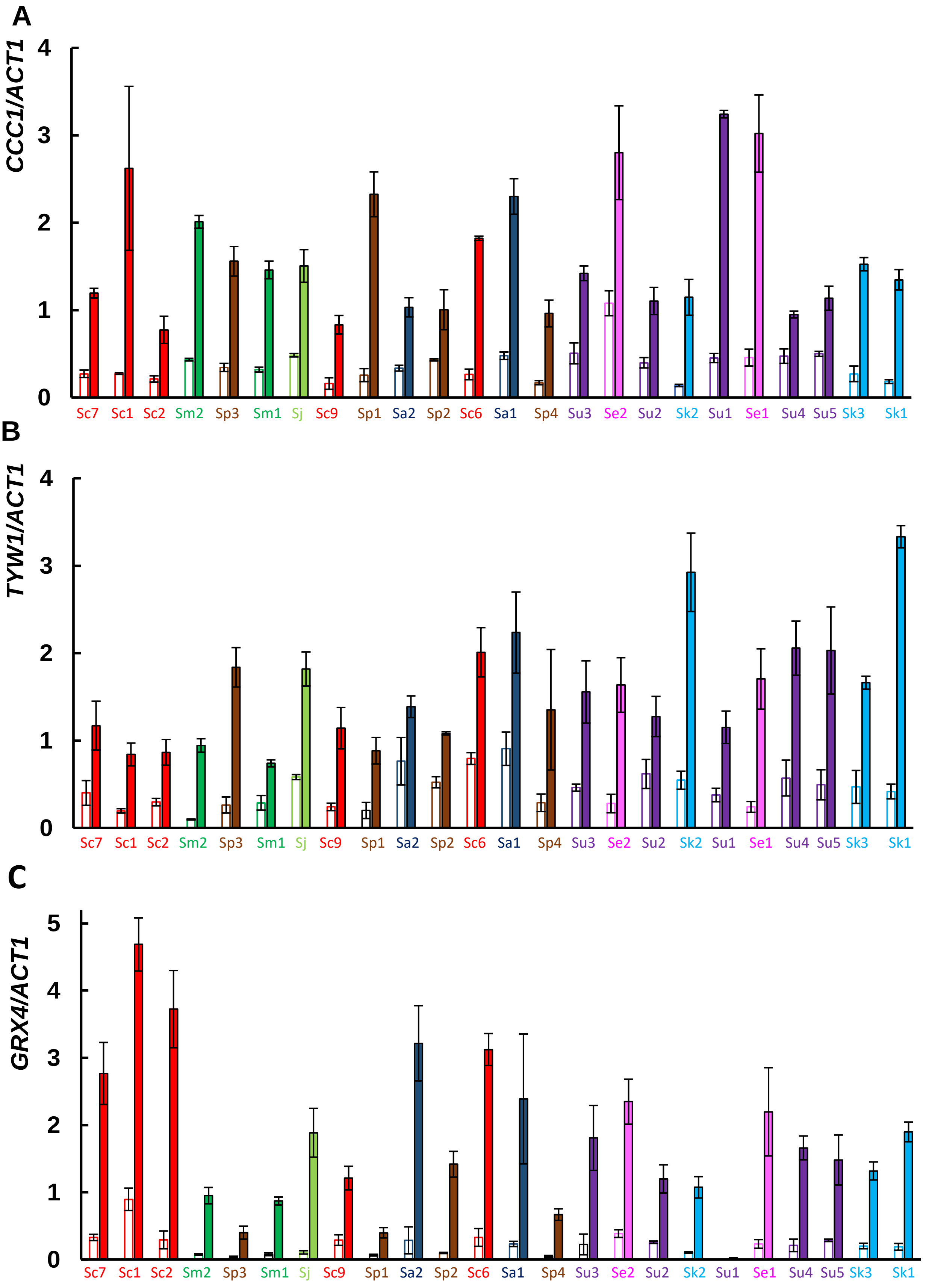


**Figure S4. Effect of iron on Yap5 targets expression in *Saccharomyces* strains.** Yeast strains were exposed to 2 mM FAS for 1 h, as described in Materials and Methods, and *CCC1* (A), *TYW1* (B), *and GRX4* (C) mRNA levels were determined by RT-qPCR. Bar plots represent the mRNA levels of each gene in normal conditions (color-bordered and unfilled bars) and in 2mM FAS (black-bordered and color-filled bars), relative to *ACT1* mRNA levels and normalized to the average of all referenced mRNA levels of the same gene . Strains are sorted based on MIC values and species-specific colors are used as in the above figures. Shown values are the average of 3 replicates and error bars constitute the standard deviation.

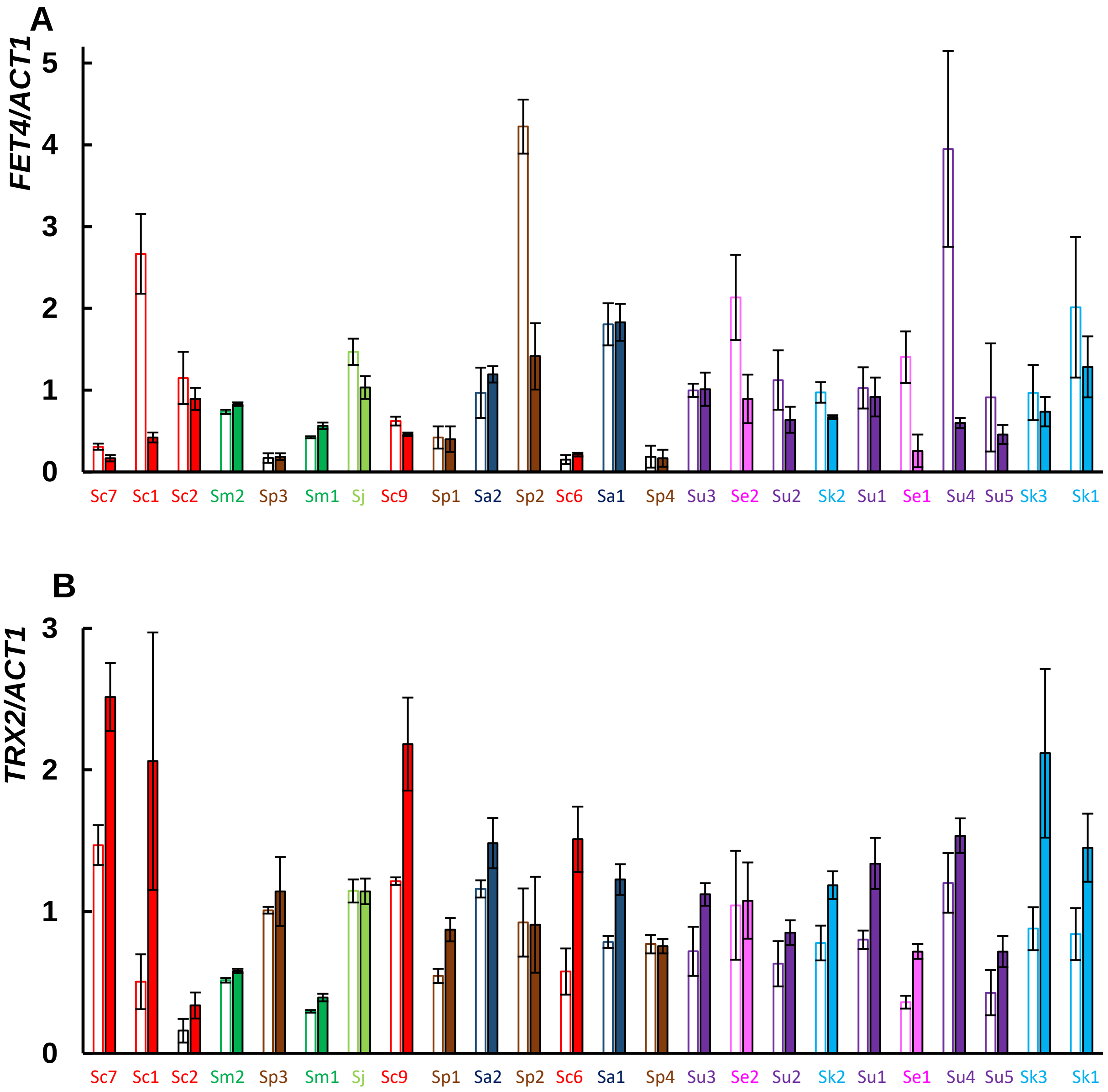


**Figure S5. Effect of iron on *FET4* and *TRX2* expression in *Saccharomyces* strains.** Yeast strains were exposed to 2 mM FAS for 1 h, as described in Materials and Methods, and *FET4* (A) and TRX2 (B) mRNA levels were determined by RT-qPCR. Bar plots represent the relative mRNA levels of each gene in normal conditions (color-bordered and unfilled bars) and in 2mM FAS (black-bordered and color-filled bars). mRNA levels of each gene are referenced to *ACT1* mRNA levels and normalized to the average of all referenced mRNA levels of the same gene. Strains are sorted based on MIC values and species-specific colors are used as in the above figures. Shown values are the average of 3 replicates and error bars constitute the standard deviation.

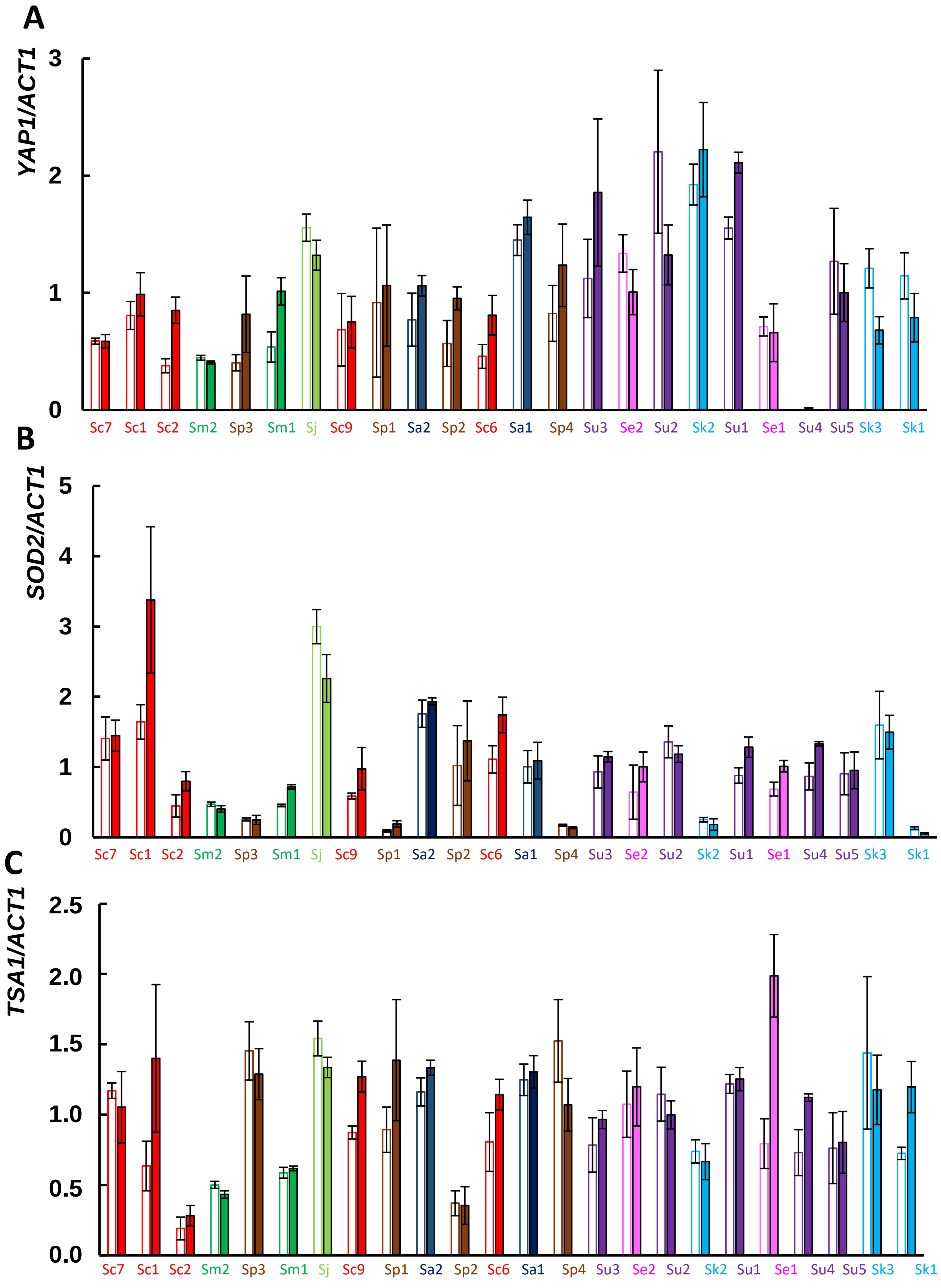


**Figure S6. Effect of iron on *YAP1*, *SOD2*, and *TSA1* expression in *Saccharomyces* strains.** Yeast strains were exposed to 2 mM FAS for 1 h, as described in Materials and Methods, and *YAP1* (A), *SOD2*, and *TSA1* (C) mRNA levels were determined by RT-qPCR. Bar plots represent the relative mRNA levels of each gene in normal conditions (color-bordered and unfilled bars) and in 2mM FAS (black-bordered and color-filled bars). mRNA levels of each gene are referenced to *ACT1* mRNA levels and normalized to the average of all referenced mRNA levels of the same gene. Strains are sorted based on MIC values and species-specific colors are used as in the above figures. Shown values are the average of 3 replicates and error bars constitute the standard deviation.

**A**

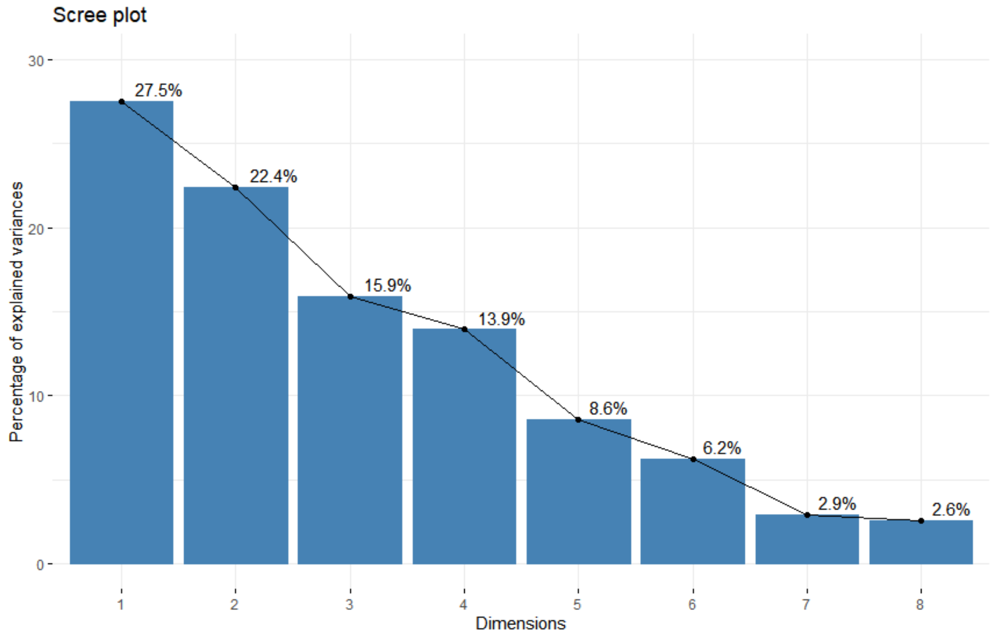


**B**

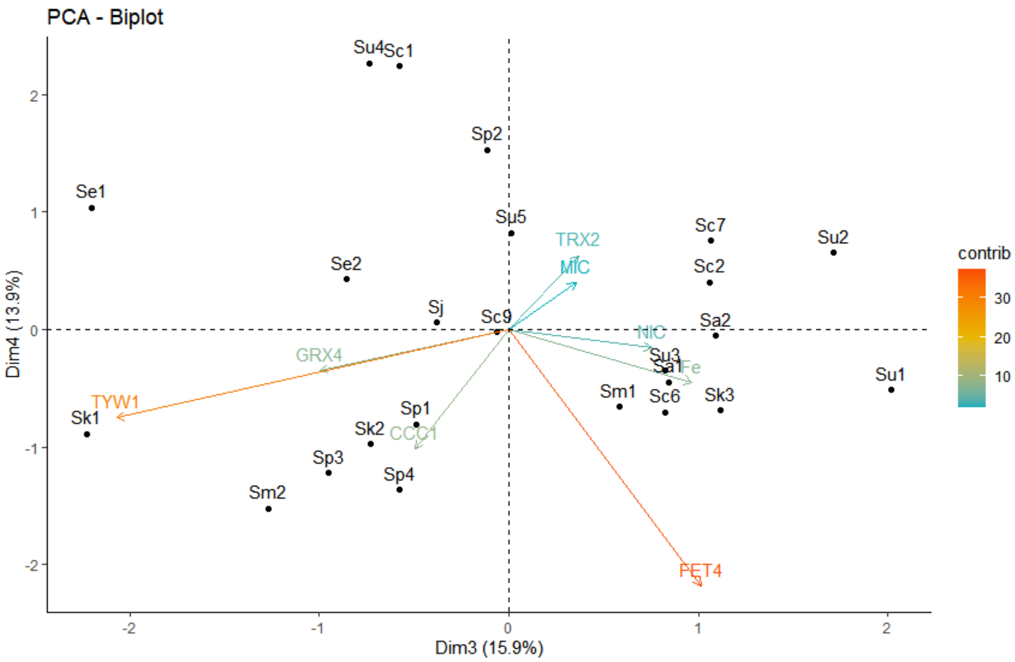


**Figure S7: Principal component analysis (PCA) of iron sensitivity, iron content, and gene expression**. A) Scree plot of the eigenvalues against the component number. The X-axis represents the different dimensions or principal components (PC) obtained in the analysis and Y-axis shows the percentage of data variance retained by each PC. The black line depicts the fraction of the total variance among the eight components. B) Two-dimensional PCA biplot of the principal components PC3 and PC4. The contribution of each variable to variance is represented by the arrow length of the vectors and the color according to the legend "contrib". Orange color describes the highest percentage of contribution to PCs and blue color the lowest. Strains (individuals) are depicted by the abbreviations used in previous figures.
