# Supplementary material for: Adaptation of Saccharomyces Species to High-Iron Conditions
Source: Int J Mol Sci. 2022 Nov 12;23(22):13965. doi: 10.3390/ijms232213965 (PMC9693265; doi:10.3390/ijms232213965)
Supplement: Supplementary file 1 [file ijms-23-13965-s001.zip › Table S3 v2 final copia.pdf]

Table S3. Summary of the results obtained in this work.

Species[a]	NIC (mM)	MIC (mM)	ΔMIC-NIC (mM)	Growth in iron (solid medium)[b]	Iron content (mg Fe/g d.w.)[d]	*CCC1* (log_2FC)[e]	*GRX4* (log_2FC)	*TYW1* (log_2FC)	*TRX2*(log_2FC)	*FET4* (log_2FC)
Sc1	3.7 ± 0.3	11.2 ± 2.2	7.5	=	1.8 ± 0.3	3.3	2.4	2.1	2.0	-2.7
Sc2	5.9 ± 0.0	9.9 ± 0.4	4.0	++	1.2 ± 0.3	1.9	3.4	1.5	1.1	-0.4
Sc3	5.5 ± 0.4	10.9 ± 0.6	5.4	+	n.d.	n.d.	n.d.	n.d.	n.d.	n.d.
Sc4	3.7 ± 0.3	7.6 ± 0.3	3.9	+	n.d.	n.d.	n.d.	n.d.	n.d.	n.d.
Sc5	4.4 ± 0.1	6.4 ± 0.2	2.1	--	n.d.	n.d.	n.d.	n.d.	n.d.	n.d.
Sc6	3.6 ± 0.1	6.5 ± 0.3	2.8	++	1.8 ± 0.3	2.8.	3.3.	1.3	1.4	0.5
Sc7	6.0 ± 0.9	11.6 ± 0.8	5.6	--	1.2± 0.1	2.1	3.1	1.5	0.8	-0.9
Sc8	3.5 ± 0.2	11.2 ± 0.2	7.7	++	n.d.	n.d.	n.d.	n.d.	n.d.	n.d.
Sc9	3.8 ± 0.2	7.5 ± 0.1	3.7	n.d.[c]	1.8 ± 0.3	2.4	2.1.	2.2	0.9	-0.4
Sp1	3.6 ± 0.3	7.4 ± 0.6	3.8	=	1.2 ± 0.5	3.2	2.6	2.1	0.7	-0.1
Sp2	3.6 ± 0.1	6.9 ± 0.1	3.3	-	1.4 ± 0.1	1.2	3.8	1.1	-0.0	-1.6
Sp3	4.2 ± 0.4	8.3 ± 0.3	4.1	-	2.2 ± 0.1	2.2	3.6	2.8	0.2	0.1
Sp4	3.8 ± 0.1	5.5 ± 0.3	1.7	--	4.5 ± 1.3	2.5	3.9	2.2	-0.0	-0.2
Sj	3.8 ± 0.2	7.6 ± 0.3	3.9	-	1.5 ± 0.4	1.6	4.1	1.6	0.0	-0.5
Sm1	3.9 ± 0.2	7.7 ± 0.1	3.8	-	1.2 ± 0.2	2.2	3.5	1.4	0.4	0.4
Sm2	4.7 ± 0.3	8.5 ± 0.3	3.9	=	2.0 ± 0.3	2.2	3.6	3.3	0.2	0.2
Sk1	3.2 ± 1.1	6.5 ± 2.6	3.4	--	1.2 ± 0.3	2.9	3.3	3.0	0.8	-0.7
Sk2	3.0 ± 0.1	4.9 ± 0.3	1.9	--	1.8 ± 0.5	3.1	3.4	2.4	0.6	-0.5
Sk3	3.2 ± 0.2	3.6 ± 0.1	0.4	---	7.1 ± 2.0	2.5	2.7	3.5	1.3	-0.4
Sa1	4.1 ± 0.3	6.3 ± 0.1-0.4	2.2	=	2.4 ± 0.6	2.3	3.4	1.3	0.6	0.0
Sa2	3.8 ± 0.2	7.2 ± 0.2	3.4	--	1.2 ± 0.3	1.6	3.5	0.9	0.4	0.3
Se1	3.6 ± 0.3	4.7 ± 0.4	1.1	--	1.5 ± 0.2	2.7	3.2	2.8	1.0	-2.5
Se2	3.7 ± 0.2	5.0 ± 0.0	1.3	+	3.4 ± 1.4	1.4	2.6	2.5	0.1	-1.3
Su1	3.8 ± 0.2	4.9 ± 0.1	1.1	--	4.0 ± 1.5	2.8	0.0	1.6	0.7	-0.2
Su2	4.1 ± 0.1	5.0 ± 0.0	0.9	-	4.8 ± 0.9	1.5	2.2	1.0	0.4	-0.8
Su3	3.9 ± 0.3	5.1 ± 0.3	1.3	--	3.5 ± 1.0	1.5	3.0	1.8	0.6	0.0
Su4	3.2 ± 0.1	4.4 ± 0.4	1.2	-	2.7 ± 0.6	1.0	3.0	1.9	0.4	-2.7
Su5	3.5 ± 0.0	3.9 ± 0.2	0.4	---	2.2 ± 0.7	1.2	2.4	2.0	0.8	-1.0

[a] Names are abbreviated as in Table 1

[b] Growth compared to that of Sc1, used as a control: =, similar; + and ++, levels of increased growth, -,-- and ---, levels of decreased growth

[c] n.d., not determined

[d] d.w., dry weight

[e] FC, fold change of expression
